# The copepod *Calanus spp*. (Calanidae) is repelled by polarized light

**DOI:** 10.1038/srep35891

**Published:** 2016-10-20

**Authors:** Amit Lerner, Howard I. Browman

**Affiliations:** 1 Department of Life Sciences, Eilat Campus, Ben Gurion University of the Negev, Beer-Sheva, 84105, Israel; 2 Institute of Marine Research, Austevoll Research Station, N-5392 Storebø, Norway

## Abstract

Both attraction and repulsion from linearly polarized light have been observed in zooplankton. A dichotomous choice experiment, consisting of plankton light traps deployed in natural waters at a depth of 30 m that projected either polarized or unpolarized light of the same intensity, was used to test the hypothesis that the North Atlantic copepod, *Calanus spp*., is linearly polarotactic. In addition, the transparency of these copepods, as they might be seen by polarization insensitive vs. sensitive visual systems, was measured. *Calanus spp*. exhibited negative polarotaxis with a preference ratio of 1.9:1. Their transparency decreased from 80% to 20% to 30% in the unpolarized, partially polarized, and electric (*e*-) vector orientation domains respectively - that is, these copepods would appear opaque and conspicuous to a polarization-sensitive viewer looking at them under conditions rich in polarized light. Since the only difference between the two plankton traps was the polarization cue, we conclude that *Calanus spp*. are polarization sensitive and exhibit negative polarotaxis at low light intensities (albeit well within the sensitivity range reported for copepods). We hypothesize that *Calanus spp.* can use polarization vision to reduce their risk of predation by polarization-sensitive predators and suggest that this be tested in future experiments.

Light in natural waters is partially linearly polarized by refraction at the surface and by scattering by water molecules and suspended matter such as sand, minerals, zooplankton and phytoplankton^1^,^2^. Very near the surface, partial linear polarization can reach maximum levels of 50–60%, both inside and outside of Snell’s window, decreasing to a maximum of 40% at depths >100 m along some lines of sight^3^,^4^,^5^. Although very little data are available on the percentage of light that is polarized in different water types, in eutrophic waters, where turbidity is moderate to high, partial polarization can be low because of multiple scattering by the high concentrations of suspended particles in the water^2^,^6^,^7^. Given the ubiquity of polarized light in water, it is not surprising that more than 70 species of aquatic animals are known to perceive it (reviewed in^3^). The possible ecological relevance of polarization vision to aquatic organisms includes habitat selection (e.g. sites for oviposition)^8^, intraspecific communication and signaling^9^,^10^,^11^,^12^,^13^, orientation and navigation^14^,^15^,^16^,^17^,^18^, and improving the detection of objects such as prey or predators through contrast enhancement^6^,^19^,^20^,^21^,^22^.

Very few studies on polarization vision have focused on zooplankton. The freshwater Cladoceran, *Daphnia pulex*, is positively polarotactic (i.e. is attracted to polarized light), regardless of light intensity, and oriented under polarized green and red light even at partial polarization levels as low as 20%^18^. *D. pulex* exhibit an escape response from shore to open water that is guided by an increase in polarization with distance from shore^23^. This was hypothesized to be a “shore flight” response to avoid shallow waters that are rich with predators. The marine copepod, *Pontella karachiensis* (Pontellidae), is negatively polarotactic (repelled from polarized light) at low light intensities (10^–7^ μE cm^−2^ nm^−1^ s^−1^), but becomes positively polarotactic at light intensities above 10^–6^ μE cm^−2^ nm^−1^ s^−1 ^24.

It has been hypothesized that the transparency of zooplankton is compromised when they are viewed against a polarized background by a polarization-sensitive viewer^25^. This occurs because the birefringent nature of the animal’s tissues alters the partial polarization of the background light passing through them^25^. As a result, the animal’s transparency decreases in the polarization domain in comparison to the intensity (unpolarized) domain - and they will be visible to a polarization-sensitive viewer. Polarization improves the detection of transparent planktonic prey by polarization-sensitive planktivores such as squid and fish under laboratory conditions^19^,^20^. In natural conditions, it has been hypothesized that polarization improves target visibility by blocking the horizontal component of the polarized radiance of the background and the visual pathway between the target and the observer^6^. Thus, transparent or partially transparent zooplankton might be either negatively or positively polarotactic: while attraction to polarized light might increase the probability of them encountering conspecifics, repulsion from polarized light might decrease their probability of being detected by polarization-sensitive planktivores. The objective of this study was to test the hypothesis that the North Atlantic copepod *Calanus spp.*, a keystone prey species in North Atlantic ecosystems^26^,^27^, responds to polarized light and displays either positive or negative polarotaxis.

## Results

**Figure 1:**
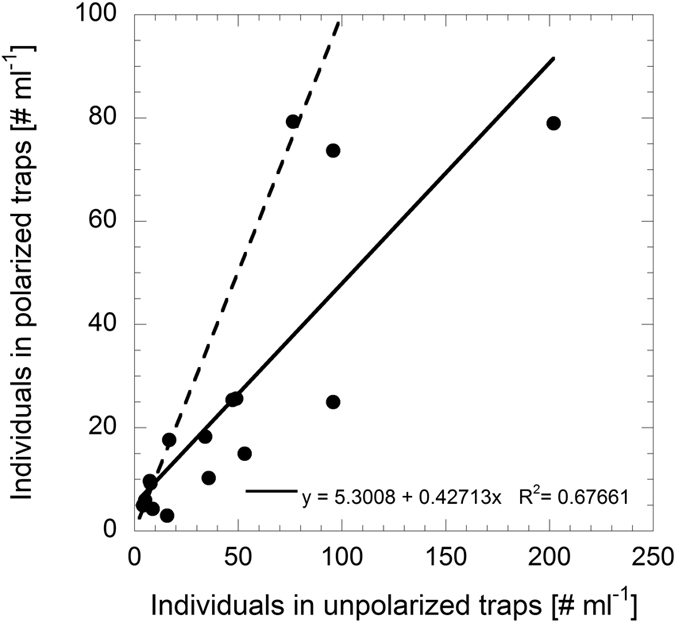
Number of individuals of *Calanus spp*. collected by polarized vs. unpolarized light traps. The dashed line represents the y = x = identity, and the solid line represents the linear regression model. These lines are presented for illustrative purposes only, to make it easier to assess the relative number of individuals collected by each trap type.

More *Calanus spp*. were attracted to the unpolarized traps than to the polarized traps by a ratio of 1.9 ± 0.3 (mean ± s.d.). This ratio differs significantly from 1 (y = x = identity) (One sample T-test; T_14,0.05_ = 2.987, *p* = 0.010; data was arcsine transformed) (Fig. 1).

**Figure 2:**
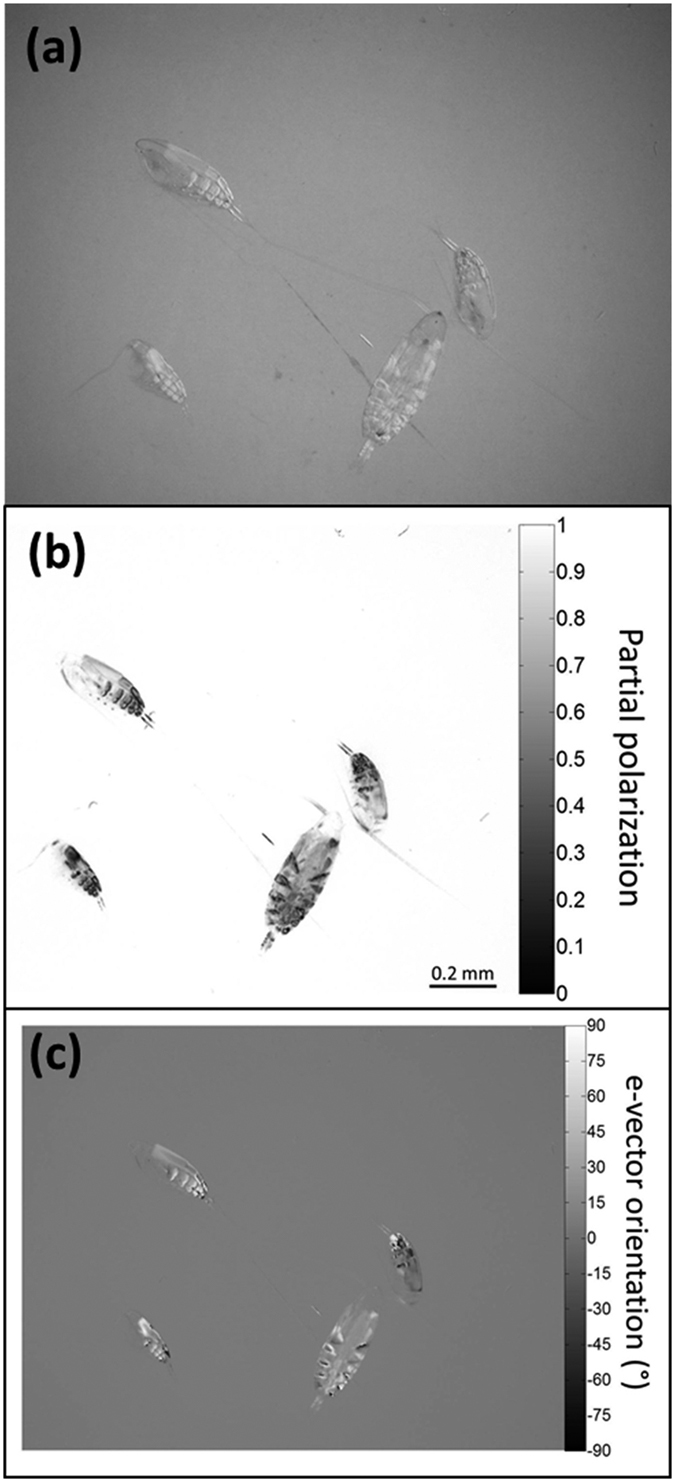
Polarization imagery of *Calanus spp.* in the green channel. (**a**) Unpolarized, (**b**) partial polarization, and (**c**) *e*-vector orientation. In (**b**) each pixel includes partial polarization value ranging between 0 (unpolarized) and 1(100% polarized). In (**c**) each pixel includes orientation value ranging between ±90° in respect to the horizon (0° orientation means horizontal, and ±90° means vertical).

The mean ± s.e. animal maximum transparency in the unpolarized domain was 0.81 ± 0.02, in the partial polarization domain 0.20 ± 0.03, and in the *e*-vector orientation domain 0.30 ± 0.05 (n = 5 pairs for each domain) (Fig. 2).

## Discussion

We tested the hypothesis that *Calanus spp*. respond too polarized light, and whether they are attracted or repelled by it (positive or negative polarotaxis). *Calanus spp.* displayed negative polarotaxis. This finding is consistent with the laboratory-based observations of Manor *et al*.^24^ on *Pontella karachiensis*, at least at the low light intensity level that was used in one of their experiments. They reported that the negative polarotactic response of *P. karachiensis* changed to positive polarotaxis at high light intensities (>0.5 × 10^−6^ μE cm^−2^ s^−1^ nm^−1^). Reducing the degree to which the light was polarized decreased the strength of the negative polarotactic response (as measured by the fraction of the animals attracted). Zooplankton are typically active at light intensities of between 10^−11^ and 10^−9^ μE cm^−2^ nm^−1^ s^−1^ 28. Thus, the change to a positive polarotaxis reported by Manor *et al*. was observed at light intensities much higher than copepods typically operate under - it is possible, therefore, that this response was actually a positive phototaxis rather than a positive polarotaxis. Therefore, we qualify our conclusion by emphasizing that *Calanus spp*. is negatively polarotactic at the relatively low light intensities used in our field experiment (i.e., 10^−11^ μE cm^−2^ s^−1^ nm^−1^). In any case, both positive and negative polarotaxis can be adaptive for these copepods. For example, positive polarotaxis might be adaptive for copepods to find conspecifics, while negative polarotaxis (repulsion) from areas with highly polarized light might serve to reduce conspicuousness to polarization-sensitive planktivores, thereby reducing predation risk. Since the *Calanus spp.* adults vertically migrate (at depth during the day; nearer to the surface at night), it is also possible that the sign of their polarotactic behavior is modulated by depth/light intensity. This is something that could be tested in future experiments.

In our experiment, the partial polarization emitted from the traps was 68%, which is consistent with the 50–60% polarization levels measured at a few meters depth, both inside and outside Snell’s window^2^,^3^,^4^. Very little data are available on the percentage of light that is polarized in different water types. The small number of such reports suggest that up to 60% of light is polarized^3^. Although it has never been measured, the ambient partial polarization in the eutrophic waters in which we conducted our experiment, and especially at the depth at which it was conducted, are not expected to exceed 40% due to multiple scattering by suspended particles in the water column. Therefore, our conclusion would be most valid for *Calanus spp.* when they are operating in highly polarized conditions, for example, close to the surface. However, as reported by Manor *et al*.^24^, copepods exhibit negative polarotaxis at polarization values as low as 30%, a level that is likely present in the waters in which we conducted our experiment. Further, the genus *Calanus* is widely distributed in the North Atlantic - occurring in a variety of water types - and is also found in the clear ultra-oligotrophic waters of the east Mediterranean sea and the Gulf of Aqaba (e.g. *C. minor*)^29^,^30^,^31^ where 60% polarization was measured in surface waters^3^. Therefore, we contend that our experiment is realistic in the context of the ecology of this genus.

The high transparency of *Calanus spp.* in the unpolarized (intensity) domain is greatly reduced in the polarization domain; that is, under some viewing conditions, they would be conspicuous to polarization-sensitive predators. This reduction in transparency in the polarization domain is consistent with the observation of 92% polarization contrast (equivalent to 8% transparency in the polarization domain) in the copepod *Undinula vulgaris*^25^ and with the hypothesis that transparent zooplankton avoid a highly polarized background as a mechanism to reduce predation by polarization-sensitive predators.

In aquatic environments, contrast enhancement of transparent prey by polarized light has only been demonstrated in laboratory experiments. Adults of the polarization-sensitive squid, *Loligo pealei*, preferred polarization active beads over polarization inactive ones, and juvenile squids tested with live zooplankton increased their detection distance to the prey by 70% under highly polarized illumination^20^. The rainbow trout, *Oncorhynchus mykiss*, located transparent prey more easily under highly polarized backgrounds^19^. However, the viewing conditions may not be the same in nature. Johnsen *et al*.^6^ measured the polarization from transparent zooplankton *in situ* and concluded that the contrast of these particles in the polarization domain, caused by forward scattering of the background light through the animal’s birefringent tissues, is not greater than their radiance contrast (as it is typically photographed and calculated in the laboratory, i.e., what is presented here in Fig. 2). Instead, polarization enhances the radiance contrast by reflection of the unpolarized downwelling radiance of the animal towards the viewer, which is much stronger than the background light. They concluded that target contrast would be enhanced by polarization through blocking the horizontally oriented background and path light by photoreceptor cells in the visual system of predators that are fixed in vertical alignment as in cephalopods^32^,^33^ or by rotating the whole eye to achieve vertical alignment as in stomatopods that are polarization sensitive^34^,^35^. Therefore, *in situ*, polarization vision may enhance contrast through reflection, regardless of the target transparency. In the case of the adult *Calanus spp.* studied here - they are active under low light conditions, far weaker than the levels of Johnsen *et al*.‘s analysis, since they spend the day at depth and migrate into the near surface waters at dusk. Under these conditions, and in contrast to Johnsen *et al*.^6^, the downwelling light is on the same order of magnitude as the background light and polarization-sensitive predators could use polarization vision to break the transparency of *Calanus spp*., as indicated here (Fig. 2). If polarization is indeed used by planktivores to enhance detection of prey, it would represent a strong driver on prey to adapt mechanisms to avoid environments that are rich in polarized light, i.e. negative polarotaxis. The observations reported here are consistent with this hypothesis, but require further research on a wider range of zooplankton taxonomic groups. Future research should also test if the degree of avoidance from areas with higher levels of background polarized light is correlated with transparency in the unpolarized light domain, which will resolve the mechanism for contrast enhancement by polarization in water.

## Methods

### *In situ* polarotaxis experiment

**Figure 3:**
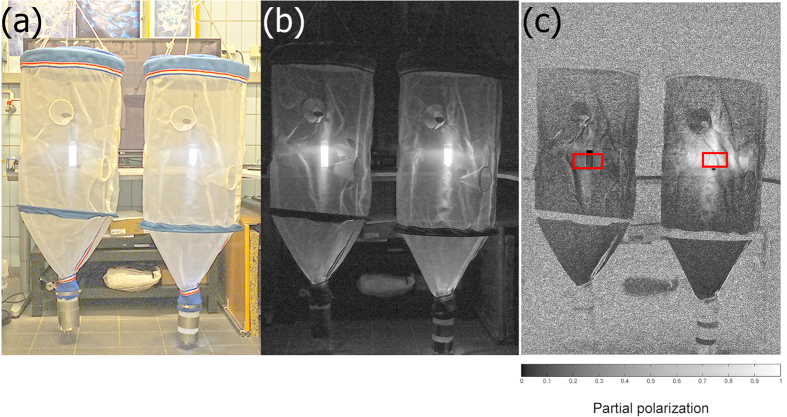
Light traps. (**a**) Red (R), green (G), blue (B) image under fluorescent lamp room light, (**b**) RGB image under dark conditions without a polarizing filter on the camera (polarization insensitive system), and (**c**) partial linear polarization image in the G channel generated from two images taken through a polarizing filter (polarization sensitive system) at vertical and horizontal orientations. In (**c**), white represents 100% polarization (fully polarized; 1 in the greyscale bar below the image) and black represents 0% polarization (unpolarized, 0 in the greyscale bar). The values at each pixel were calculated using equation (1). The red rectangles in (**c**) represent the pixels sampled for the statistical analysis presented in Table 1. The polarization orientation emitted by the two traps was horizontal.

To test for polarotaxis in *Calanus spp.*, paired sets of plankton light traps (Bellamare, USA) (Fig. 3) were deployed during the period 4–13 April 2013 (n = 15 paired sets; each set was considered a replicate), between 23h00 and 04h00 from the end of the dock at the Institute of Marine Research, Austevoll Research Station, Austevoll, Norway (60.086N, 5.262E). The light traps were deployed for 90 min. at a depth of 30 m and the distance between the traps was 2 m. The mesh size of the trap itself was 355 μm and the codend mesh size was 300 μm. To control for any effect of current on the collections, the trap locations were randomly alternated between replicates.

Two species of *Calanus* that are abundant in this area, *C. finmarchicus* and *C. helgolandicus* (typically at a ratio of 9:1), were collected in the light traps in large numbers. Since the individuals were not identified to the species level, they are referred to here at the genus level only. These two species of *Calanus* are very similar morphologically and, most importantly in the context of this study, there is no reason to expect them to behave differently with respect to their sensitivity or response to polarized light since the mechanism for polarization perception, an orthogonal arrangement of microvilli in which the retinal molecules that absorb light are embedded, is common and well characterized in arthropods^36^ and has also been reported in the eye of the copepod *Pontella karachiensis*, Pontellidae^24^.

The paired light traps were outfitted with identical battery-powered submersible LED flashlights that projected polarized or unpolarized light. The polarized light source was wrapped with a diffusing sheet (SKU 25049-01.007′, Inventables, Inc., IL, USA) and then a polarizing sheet oriented horizontally (Linear polarizers, PF-006 (6 mm), Aflash Photonics Ltd., TX, USA), and the unpolarized light source was wrapped with the same sheets arranged in the opposite order and with the same horizontal orientation, thus ensuring that the light sources in the paired traps projected the same light intensity. The flashlights were randomly switched between the traps to limit possible effects of trap structure or light positioning on capture probability. The light intensity and polarization spectra of the light sources were measured (400–700 nm) at 1 nm intervals using a spectrometer and optical fiber (ADC-1000-USB and UV/VIS600 Ocean Optics, Dunedin, Florida, USA; for details see ref. 8). The transmission of the polarizing and diffusing sheets did not vary with wavelength across the range measured. The mean ± s.d. intensity (420–700 nm) of the light sources in the paired traps was 0.7 ± 0.4 and 1.2 ± 0.5 × 10^–11^ μE cm^−2^ nm^−1^ s^−1^ for the unpolarized vs. polarized traps respectively.

To quantify the differences in polarization between the two traps, polarized images were generated using a tripod-mounted Fujifilm Corporation X-S1 camera (Fig. 3). An unpolarized (intensity) image was taken without a polarizing filter on the camera. The polarized image, in which each pixel includes a partial polarization value, was generated by taking two images through a polarizing filter on the camera with vertical or horizontal orientations and calculating the partial polarization value at each pixel, *P*, using the equation


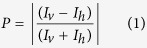


where, *I*_*v*_ and *I*_*h*_ are the pixel values of the images taken at vertical vs. horizontal orientations respectively. While the two traps could not be distinguished by their reflected intensity, the traps projected linearly horizontally polarized and unpolarized light (mean ± s.d. partial polarization, *P* = 68.1 ± 11.4% and 15.4 ± 7.9%, respectively, 2-sample Ttest, T_(55384,0.05)_ = 638.08, *p* < 0.0001) (Fig. 3), while the partial polarization emitted from the polarizing trap did not vary between the RGB channels (One-way ANOVA, the statistical details are summarized in Table 1).

The partial polarization of a point source of light, or of a target through which light is transmitted, attenuates by 30–60% over distances of few meters, depending on water clarity^37^. At distances of centimeters, the target is only depolarized by <10%. Therefore, a copepod that approached the two light traps from a distance of many meters would see two unpolarized targets of the same intensity (because the polarized light emanating from the polarized trap has been attenuated). However, as the copepod gets closer to the trap, the difference between their partial polarization increases and, at some point, becomes detectable. It is at that point that the copepod would be able to make a choice between the two traps, and this is what we tested and measured (see the next paragraph).

The number of *Calanus spp*. collected in each of the 15 paired sets of light traps was counted by sub-sampling all of the animals collected. The content of each trap was placed in a 1-L jar of sea water from which 5 sub-samples of 10 ml each were withdrawn using a W/S Hensen-Stempel pippete (Part # 3-1805-C42, Wildco, FL, USA). The number of *Calanus spp*. in each sub-sample were counted and averaged over the 5 sub-samples. The ratio of the number of individuals collected by the unpolarized trap vs. the polarized trap was calculated for each replicate. A one-sample t-test was used - because different animals were captured in each trap and the experiment was not a repeated measure design - to test whether the average of the ratios of all replicates was different than 1 (i.e. to test the null hypothesis that the number of individuals in both traps was the same). Since the ratios are proportional values (ranging between 0–1), an arcsine transformation was applied. Since the ratios were sometimes greater than 1 (due to higher number of individuals in the unpolarized trap than in the polarized trap), the ratio was divided by 10 before applying the statistical test (since only values between 0-1 can be arcsine transformed) and thereafter back-transformed. The average and s.d. of the ratios were calculated on the transformed data, and then reverse transformed to obtain their actual values.

### Polarization imagery and transparency

To evaluate the transparency of *Calanus spp*. in the unpolarized and polarization domains, live individuals were gently tranquilized using one drop of clove oil and placed between two linear polarizers under a binocular microscope outfitted with a digital camera. Unpolarized and polarized images of the animals were generated (Fig. 2; for details see ref. 25). The polarization images included two photographs, one for the partial polarization, in which the pixel value ranged between 0–100% (unpolarized-fully polarized), and a second photograph for the *e*-vector orientation in which the pixel value ranged between −90° and 90° (with respect to horizontal orientation). To evaluate the maximum transparency of *Calanus spp*. in the partial polarization (*T*_*P*_), and in the *e*-vector orientation (*T*_*ψ*_) domains in comparison to the unpolarized domain (*T*_*up*_), pairs of pixel values *v* from the animal body and adjacent background were measured. The transparency *T* was then calculated by:


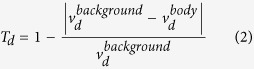


where *d* is the light domain *up* (unpolarized)*, P* (partial polarization), or *ψ (e*-vector orientation), and *v* is the pixel value. For *T*_*ψ*_ the difference between the body and the background was normalized by 90°, the maximum value of the difference, as the background value was set to zero (horizontal orientation) hence could not be divided by. For this analysis, only the most conspicuous body parts were chosen (*P* < 0.3; *ψ* > |60°|) *-* as a measure of the minimum level of transparency (*sensu*^25^).

## Additional Information

**How to cite this article**: Lerner, A. and Browman, H. I. The copepod *Calanus spp*. (Calanidae) is repelled by polarized light. *Sci. Rep.* **6**, 35891; doi: 10.1038/srep35891 (2016).

## Figures and Tables

**Table 1:** Mean ± standard deviation and one-way ANOVA of partial polarization for each of the red (R) green (G) and blue (B) channels emitted by the polarized and unpolarized traps in Fig. 1.

	R channel	G channel	B channel	F (df, α)	*p-value*
Polarized trap	0.69 ± 0.11	0.68 ± 0.11	0.67 ± 0.11	272.48 (77919, 0.05)	<0.0001
Unpolarized trap	0.15 ± 0.08	0.15 ± 0.08	0.15 ± 0.08	57.06 (77919, 0.05)	<0.0001
